# Near-infrared photoactive Ag-Zn-Ga-S-Se quantum dots for high-performance quantum dot-sensitized solar cells

**DOI:** 10.3762/bjnano.13.110

**Published:** 2022-11-14

**Authors:** Roopakala Kottayi, Ilangovan Veerappan, Ramadasse Sittaramane

**Affiliations:** 1 Department of Physics, Kanchi Mamunivar Govt. Institute for PG Studies and Research, Puducherry-605008, India

**Keywords:** alloyed QDs, photoconversion efficiency, photovoltaic performance, quantum dots

## Abstract

The high light-harvesting ability of quantum dots (QDs) plays an eminent role in the performance of solar cells. In this study, we synthesized Ag-Zn-Ga-S-Se-based alloyed QDs by colloidal hot injection and characterized them. The X-ray photoelectron spectrum analysis confirms the +1, +2, +3, −2, and −2 oxidation states of, respectively, Ag, Zn, Ga, S, and Se in the QDs, and the energy-dispersive X-ray spectrum analysis confirms the 1:1:1:1.5:1.5 stoichiometric ratio of, respectively, Ag, Zn, Ga, S, and Se. These two results indicate the formation of I-II-III-VI_3_-type alloyed crystals (AgZnGaS_1.5_Se_1.5_ nanocrystals). TEM image analysis reveals the QD nature of the synthesized Ag-Zn-Ga-S-Se nanocrystals. The X-ray diffraction pattern confirms the hexagonal structure. Due to the near-infrared light absorption capability, the synthesized QDs were used as the sensitizer to fabricate QDSCs. The fabricated QDSCs were characterized by using electrochemical impedance spectroscopy and photovoltaic performance studies. The fabricated QDSC have superior electrochemical activity with a photoconversion efficiency of 4.91%.

## Introduction

Human life depends on various forms of energy. Approximately 13 terawatts of energy are required to maintain the current lifestyle of the world’s population [1]. Our primary source of clean abundant energy is the sun. The amount of energy received from the sun is about twice of that obtained from all non-renewable resources.

Photovoltaic (PV) cells or solar cells are considered a good solution to the raising energy demands. Third-generation PV cells show advantages such as high theoretical photoconversion efficiency (PCE = 44%) [2], low cost, and flexibility. QDSCs have been considered a candidate for PV cells [3] because of the good optoelectronic properties of colloidal QDs [4–7]. At present, QDSCs have reached 14.4% efficiency. In QDSCs, QDs are added to the metal oxides. Our earlier reports demonstrated that porous TiO_2_ nanofibers possess an enormous surface area for the maximum absorption of QDs [8–10].

For the past decades, binary or ternary QDs based on lead and cadmium have been used as sensitizers in QDSCs. However, due to the high toxicity and low efficiency, research moved to group I-III-VI QDs such as Cu-In-Se, Cu-In-S, Ag-In-Se, Ag-In-S, Ag-Ga-S, and Ag-Ga-S. Even though their light-harvesting capability is still limited by a high number of surface trap state defects [11–12]. In order to minimize the number of these defects, a wide-bandgap material, such as ZnS or ZnSe, is deposited on group I-III-VI QDs. Zhang et al. over coated a ZnS layer [13–14] on Cu-In-S and Cu-In-Se QDs to obtain highly efficient sensitizers for QDSCs. Hua Zhang et al. doped Zn into AgInS_2_ and obtained Zn-Ag-In-S QDs. The QDSCs assembled by using these QDs exhibited a PCE of 4.50% [15]. The authors reported that the incorporation of zinc improved the optical properties and the PCE of AgInS_2_ QDs. Halder et al. reported the effect of doping Zn into a AgInSe_2_ host [16]. Recently, Larsen et al. reported that, due to the appropriate bandgap (1.6–1.8 eV), AgGaSe_2_ is a wide-range light absorber in thin film solar cells [17]. Tianya Bai et al. [18] examined that ZnS-coated AgGaS_2_ nanocrystals (AgGaS_2_/ZnS core–shell nanocrystals) have a tunable bandgap and PL colors. Hence, they have application potential in optoelectronic devices.

Herein, we synthesized, for the first time, Ag, Zn, Ga, S, and Se-based alloyed QDs (AZGSSe QDs) and investigated the optoelectric and morphological properties. Due to the near-infrared (NIR) light absorption, the QDs were used as sensitizers to fabricate QDSCs. The QDSC constructed by using AZGSSe QD-sensitized TiO_2_ nanofibers (AZGSSe/TiO_2_) as the photoanode showed a PCE of 4.91%.

## Experimental

### Materials

Silver(I) chloride (AgCl), selenium powder (Se), mercaptopropionic acid (MPA), zinc(II) chloride (ZnCl_2_), gallium(III) chloride (GaCl_3_), sulfur powder (S), 1-dodecanethiol (DDTh), ʟ-cysteine, copper(II) chloride, polyvinylidene fluoride (PVDF), titanium isisopropoxide (TIP), glycerol, oleylamine (OAM), titanium tetrachloride (TiCl_4_), *N*-methyl-2-pyrrolidine (NMP), chloroform, acetonitrile, ethanol, and methanol were purchased from Merck India.

### Synthesis of Ag-Zn-Ga-S-Se QDs

Ag-Zn-Ga-S-Se QDs (AZGSSe) were prepared by a colloidal hot injection method. It was done according to the following procedure: 0.048 g of S and 0.118 g of Se were mixed well with 5 mL of DDTh, and 5 mL of OAM and stirred for 4 h to form a SSe stock solution. 0.143 g of AgCl, 0.136 g of ZnCl_2_, 0.176 g of GaCl_3_, 10 mL of OAM, and 2 mL of DDTh were refluxed at 100 °C for 30 min. The temperature was gradually increased up to 200 °C and the pre-prepared SSe stock solution was rapidly injected under continuous stirring. The mixture was maintained at 200 °C for 10 min and quickly poured into cold methanol to form AZGSSe QDs. After purification purification via centrifugation, the QDs were dispersed in 5 mL chloroform.

### Preparation of a AZGSSe QD-sensitized TiO_2_ NF-based photoanode

A pre-prepared TiO_2_ nanofiber (NF) paste was coated on FTO glass via the doctor blade method, which was followed by sintering at 450 °C for 30 min as described earlier [8–10]. The glass plate was dipped in 0.12 M of titanium tetrachloride solution at 70 °C for 30 min and was then rinsed with distilled water thoroughly and dried at 60 °C. After that, the electrode was soaked for 24 h with a MPA/acetonitrile solution 3:7 (v/v). This substrate was then immersed into the colloidal AZGSSe QD solution for 48 h to obtain the AZGSSe-sensitized TiO_2_ NF-based photoanode.

### Assembly of QDSCs

Earlier reports revealed that Cu_2_S is a low-cost and efficient counter electrode (CE) for QDSCs [2,19–20]. Hence, in this work Cu_2_S was chosen as the CE material. The CE was fabricated as described in [8]. At first, Cu_2_S NPs were prepared by a hydrothermal method using ʟ-cysteine and copper(II) chloride. Then the Cu_2_S paste containing 95% of Cu_2_S and 5% of PVdF in NMP was coated onto FTO glass. This substrate was then dried at 60 °C in vacuum for 12 h to obtain the Cu_2_S-based CE. CE and photoanode were sandwiched with a 60 µm hot melt spacer at 110 °C for 50 s and clipped together. After that, the electrolyte was injected between the electrodes through pre-drilled holes in the CE to get QDSCs.

### Physical characterization

The crystalline structure and size of the synthesized QDs were examined by X-ray diffraction (Riganku Ultima IV XRD spectrometer with nickel-filtered Cu Kα radiation with a step width of 0.02°) High-resolution transmission electron microscopy was carried out on a JEOL JSM-7600F. The electronic states of the elements and their atomic ratio in the prepared samples was analyzed by using XPS (Kratos AXIS Ultra DLD) and EDX (Bruker Nano XFlash detector attached to the HRTEM). Optical properties were examined by using a UV–vis–NIR spectrophotometer (Perkin Elmer L-650 UV) and a photoluminescence spectrofluorometer (Jobin Yvon Horiba Nanolog). Impedance analysis of the fabricated QDSCs was conducted by using a CH instruments 760 A electrochemical workstation at applied frequencies of 100 kHz to 1 MHz with 10 mV AC voltage recording the Nyquist plots. The *J*–*V* curves were measured by using an AM 1.5 solar simulator (Oriel instruments 67005) of 100 mW·cm^−2^ power density. This power density was calibrated with the photocurrent of the reference cell (crystal Si capped by an IR cut filter). Fill factor (FF) and photoconversion efficiency (η) were calculated by using the standard equations reported elsewhere [21].

## Results and Discussion

### Studies of Ag-Zn-Ga-S-Se QDs

**Figure 1:**
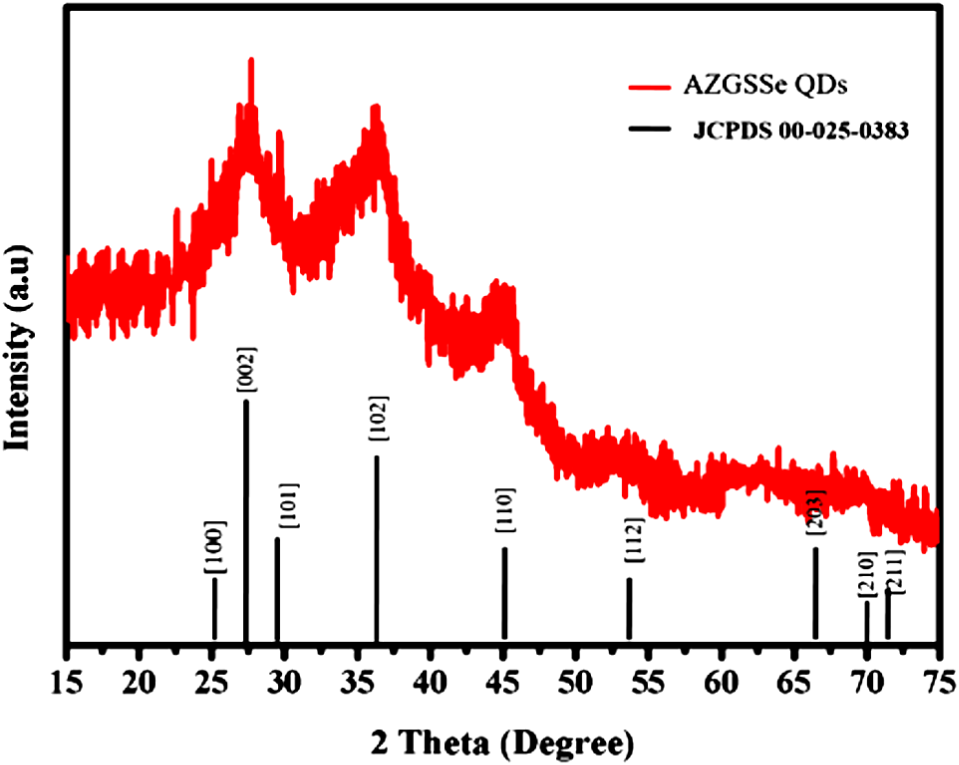
XRD pattern of AZGSSe QDs.

The synthesized Ag-Zn-Ga-S-Se (AZGSSe) QDs were analyzed using XRD. The XRD pattern (Figure 1) shows peaks at 24.9°, 26.6°, 28.3°, 36.8°, 43.9°, 54°, 64°, 67°, and 69° corresponding, respectively, to the (100), (002), (101), (102), (110), (112), (203), (210), and (211) planes of the hexagonal crystals (JCPDS: 00-025-0383). The crystallite size of these QDs was found to be 5.03 nm using the Scherrer equation [22].

**Figure 2:**
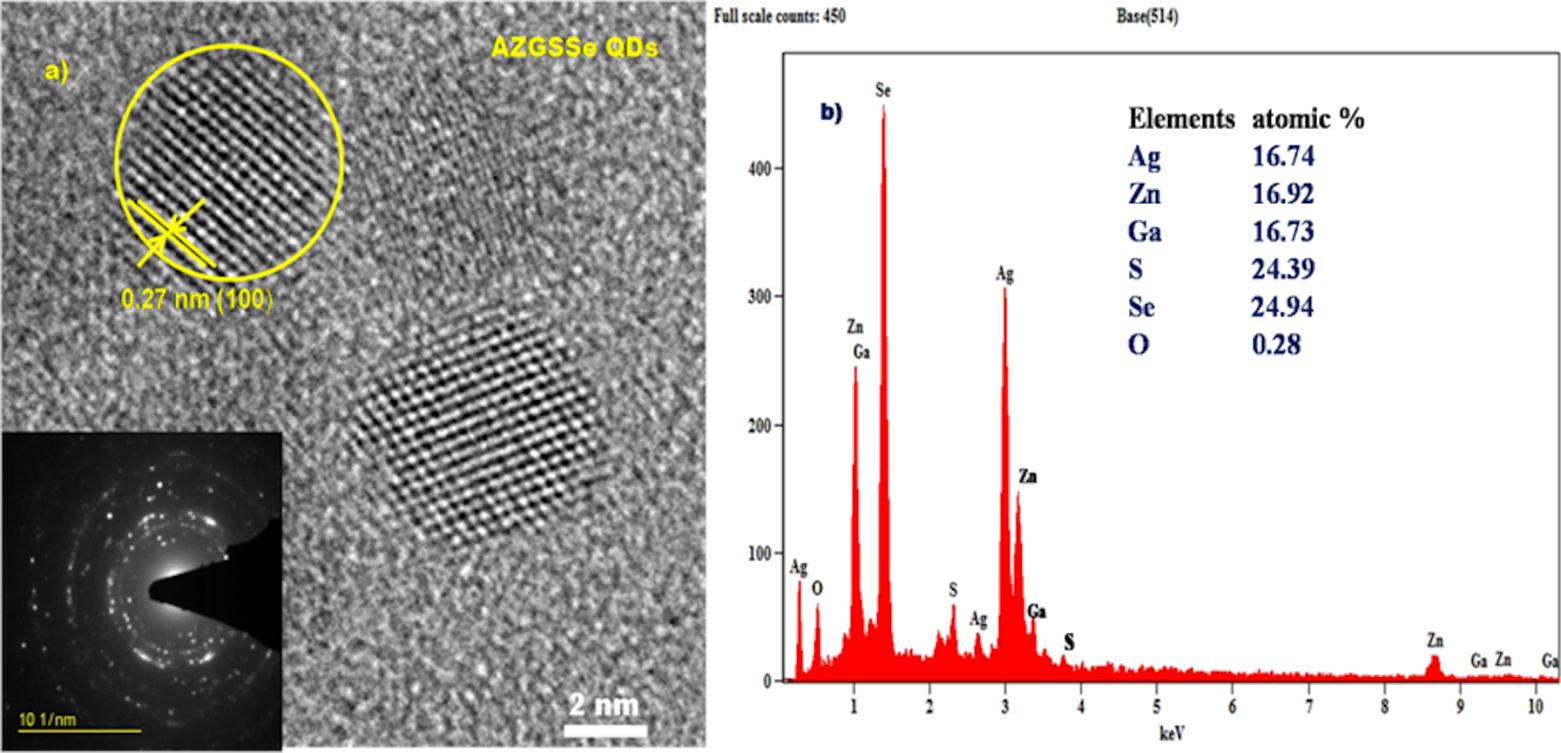
(a) HRTEM image and (b) EXD spectrum of AZGSSe QDs.

Figure 2a shows a HRTEM image of AZGSSe QDs. The average mean diameter was found to be 5.11 nm and the spacing of one of the lattice fringes was observed to be 0.27 nm. This corresponds to the spacing distance of the (100) planes of hexagonal crystals [23–25]. The inset of Figure 2a shows the SAED pattern of the synthesized AZGSSe QDs. Well-defined concentric rings in this pattern confirm the crystalline nature of AZGSSe QDs. Figure 2b shows the EDX spectrum of AZGSSe QDs. The fractions of Ag, Zn, Ga, S, and Se were found to be 16.74, 16.92, 16.73, 24.39, and 24.94 atom %, respectively. The corresponding stoichiometric ratio was calculated as 1:0.99:1.01:1.46:1.49, which is approximately a ratio of 1:1:1:1.5:1.5 , indicating the formation of AgZnGaS_1.5_Se_1.5_ QDs.

### XPS studies

**Figure 3:**
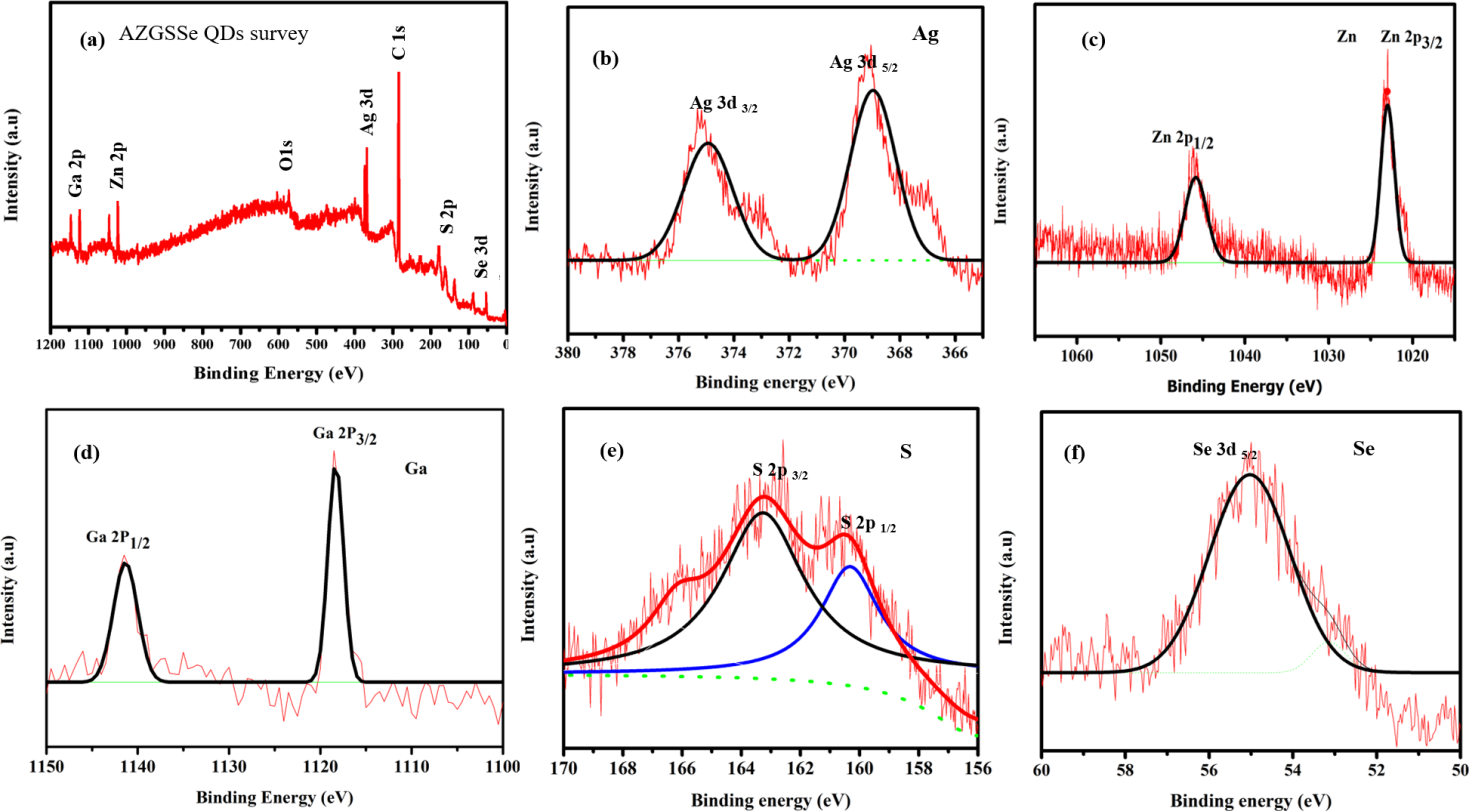
(a) Survey; (b) Ag 3d; (c) Zn 2p; (d) Ga 2p; (e) S 2p, and (f) Se 3d XPS spectra of AZGSSe QDs.

XPS spectra of AZGSSe QDs are depicted in Figure 3. To avoid the background noise, peak fits were included in all elemental spectra. Figure 3a shows Ag 3d, Zn 2p, S 2p, Se 3d, Ga 2p, C 1s, and O 1s peaks. Here, the Ag 3d, Zn 2p, S 2p, Se 3d, and Ga 2p peaks arise from AZGSSe QDs, and the O 1s and C 1s peaks are due to the absorption of oxygen and hydrocarbons from the environment [21,26–27]. The elemental Ag 3d spectrum (Figure 3b) shows the peaks of Ag 3d_5/2_ (368.32 eV) and Ag 3d_3/2_ (374.38 eV) with a splitting of 6.06 eV, indicating the existence of Ag^+^ ions [27–28]. The elemental Zn spectrum (Figure 3c) shows the peaks of Zn 2p_3/2_ (1021.82 eV) and Zn 2p_1/2_ (1044.78 eV) with a splitting of 22.96 eV, indicating the existence of Zn^2+^ ions [27–28]. The elemental Ga spectrum (Figure 3d) shows the peaks of Ga 2p_3/2_ (1116.96 eV) and Ga 2p_1/2_ (1143.80 eV) with a splitting of 26.84 eV indicating the existence of Ga^3+^ ions [27–28]. The elemental S spectrum (Figure 3e) shows the peaks of S 2p_3/2_ (161.95 eV) and S 2p_1/2_ (163.13 eV) with a splitting of 1.18 eV indicating the existence of S^2−^ ions [27,29]. The elemental Se spectrum (Figure 3f) shows the peaks of Se 3d_5/2_ (55.65 eV) indicating the existence of Se^2−^ ions [27,29] .This result confirms that the synthesized QDs contain Zn^2+^, Ag^+^, Ga^3+^, S^2−^ and Se^2−^ ions forming I-II-III-VI_3_-type AgZnGaS_1.5_Se_1.5_ QDs.

### Optical studies

**Figure 4:**
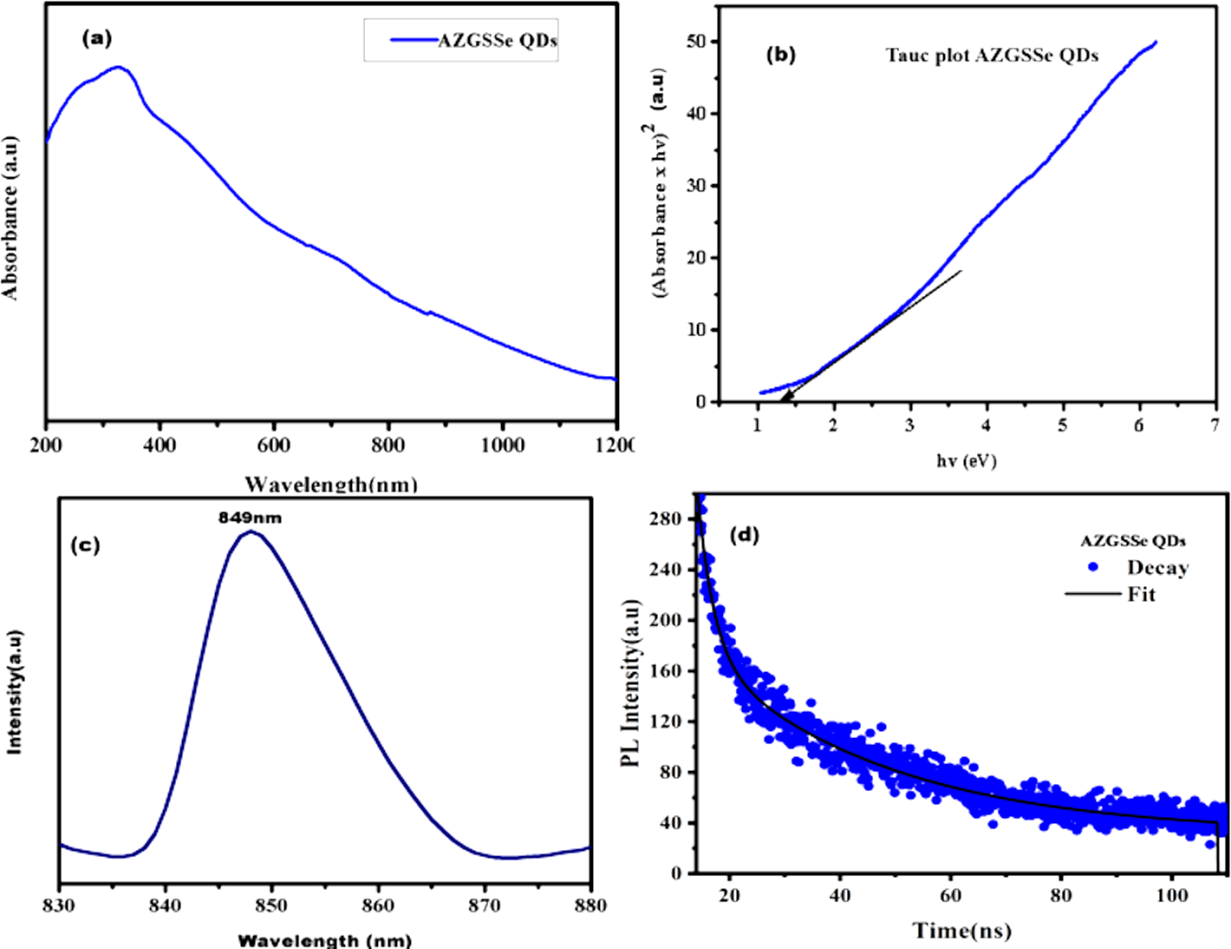
(a) UV–vis–NIR absorption spectrum; (b) Tauc Plot; (c) PL emission; (d) PL decay spectrum of AZGSSe QDs.

The UV–vis absorbance spectrum of colloidal AZGSSe QDs (Figure 4a) reveals a wide absorption range in the near-infrared (NIR) region. This confirms the NIR photoactive nature of the synthesized QDs. Figure 4b depicts the Tauc plot [30] of the synthesized QDs. From this, the bandgap energy of AZGSSe QDs was found to be 1.37 eV. This confirms that the QDS can serve as effective sensitizers.

The PL emission spectrum of AZGSSe QDs (Figure 4c) was recorded at an excitation wavelength of 295 nm. In this spectrum, a single emission peak is detected at 830 to 880 nm range with a FWHM of 20 nm. This signifies defect-free crystals [31–32]. Figure 4d shows the PL decay spectrum of AZGSSe QDs fitted with a biexponential function (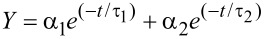), where τ_1_ and τ_2_ are the carrier life times, and α_1_ and α_2_ are the relative amplitudes of the respective carrier lifetime. The average life (τ_s_) of the AZGSSe QDs was calculated to be 42.64 ns [33].

### Studies of Ag-Zn-Ga-S-Se QD-sensitized TiO_2_ NFs

**Figure 5:**
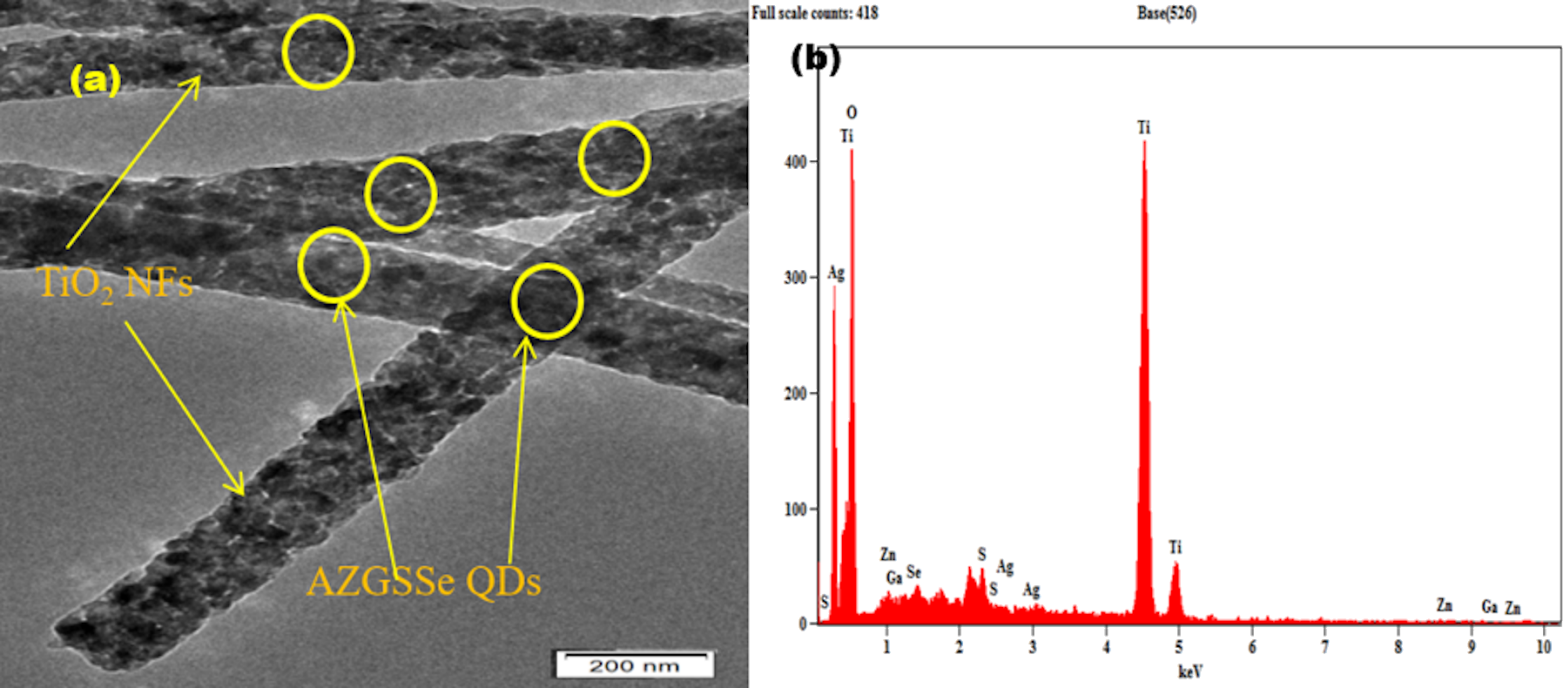
(a) HRTEM image and (b) EDX spectrum of AZGSSe QDs/TiO_2_ NFs.

The surface morphology of AZGSSe/TiO_2_ was examined through HRTEM and EDX analysis. The HRTEM image (Figure 5a) shows the presence of AZGSSe QDs on the TiO_2_ NFs. The EDX spectrum (Figure 5b) shows the peaks of Ti, O, Ag, Zn, Ga, S, and Se. These analyses prove the incorporation of AZGSSe QDs in the TiO_2_ NFs.

**Figure 6:**
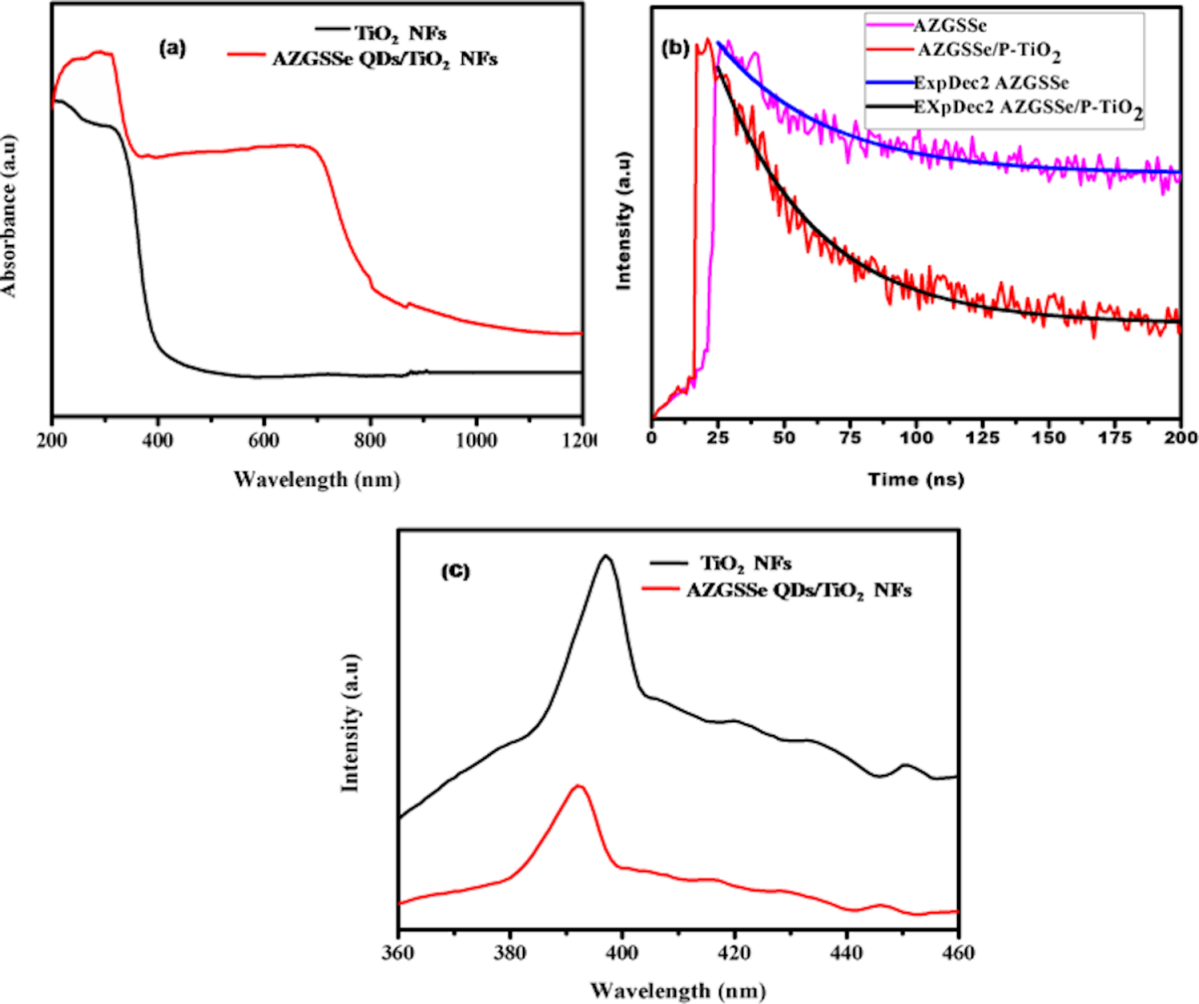
(a) UV–vis absorbance spectra; (b) PL decay spectra; (c) PL emission spectra of the AZGSSe/TiO_2_ NF-based photoanode.

The UV–vis absorption spectrum of the AZGSSe/TiO_2_-based photoanode in comparison with TiO_2_ NFs shown in Figure 6a. It can be observed that AZGSSe/TiO_2_-based photoanode has a light absorption in the NIR region. This signifies that AZGSSe/TiO_2_ is an efficient photoanode for QDSCs and it produces more electron–hole pairs, which helps to improve the photocurrent density. Time-resolved photoluminescence (TRPL) studies were carried out to evaluate the electrons decay time of AZGSSe/TiO_2_. The decay curve was fitted with a biexponential function [33] and it is shown in Figure 6b. From this, the average lifetime (τ_s_) of AZGSSe/TiO_2_ was found to be 18.92 ns. Then the rate constant (*K*_eff_) of the electron transfer is calculated to be 2.9 × 10^7^ s^−1^ from the equation:


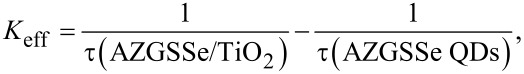


where τ(AZGSSe/TiO_2_) is the average electron lifetime of AZGSSe/TiO_2_ and τ(AZGSSe QDs) is the average electron lifetime of AZGSSe QDs [34].

Figure 6c shows the PL emission spectra of the AZGSSe/TiO_2_ NF-based photoanode in comparison with TiO_2_ NFs. It reveals that the PL intensity of AZGSSe/TiO_2_ NFs is quenched. This is due to the enhanced electron transfer from the conduction band of AZGSSe QDs to TiO_2_ NFs with minimized charge recombination rate [35–37].

### PV cell studies

**Figure 7:**
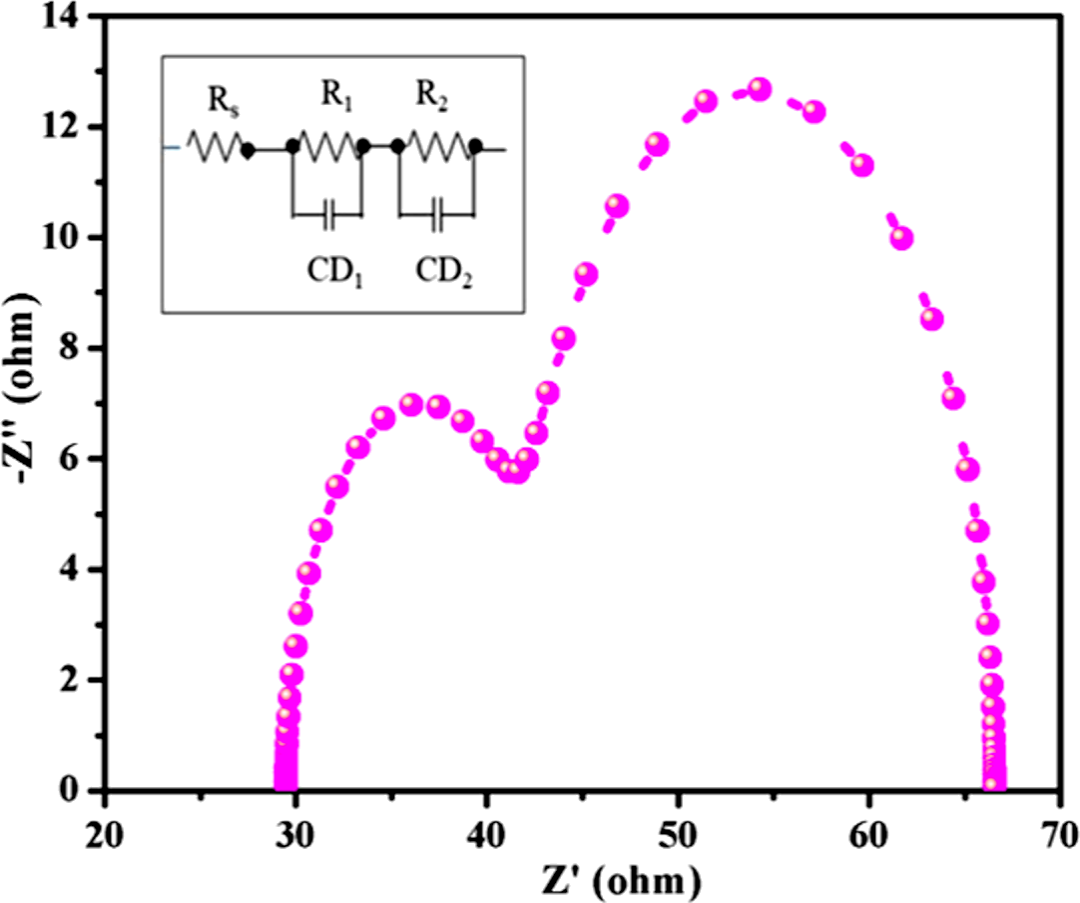
Nyquist plot of the fabricated QDSCs.

The electron transfer mechanism of the fabricated QDSC was examined by using impedance analysis. The impedance spectrum plotted in the form of a Nyquist plot (Figure 7) was fitted with the equivalent circuit shown in the inset of Figure 7. The corresponding parameters are shown in Table 1. The Nyquist plot consists of two hemispheres, namely the high-frequency region hemisphere and the low-frequency region hemisphere. The high-frequency region hemisphere is associated with the charge transfer resistance (*R*_1_) that appears at the interface of electrolyte and counter electrode. The low-frequency region hemisphere is attributed to the charge transfer resistance (*R*_2_) appearing at the interface of electrolyte and photoanode. The sheet resistance (*R*_s_) is the resistance of the intercept of the real axis. Similarly, CD1 is the double layer capacitance in the counter electrode/electrolyte interface and CD_2_ is the capacitance in the photoanode/electrolyte interface. In this work, we focused on *R*_2_, and it was observed to be 26.78 Ω. This low value of *R*_2_ reveals that the AZGSSe/TiO_2_ NF-based photoanode has superior electrochemical activity. It can boost charge recombination and transport electrons readily, which contributes to the enhanced performance of the QDSC.

**Table 1:** Electrochemical impedance (EIS) parameter values of the fabricated QDSCs.

Photoanode	*R*_S_ (Ω)	*R*_1_ (Ω)	CD_1_ (F)	*R*_2_ (Ω)	CD_2_ (F)

AZGSSe/TiO_2_	29.96	14.35	1.75 × 10^−6^	26.78	4.62 × 10^−6^

**Figure 8:**
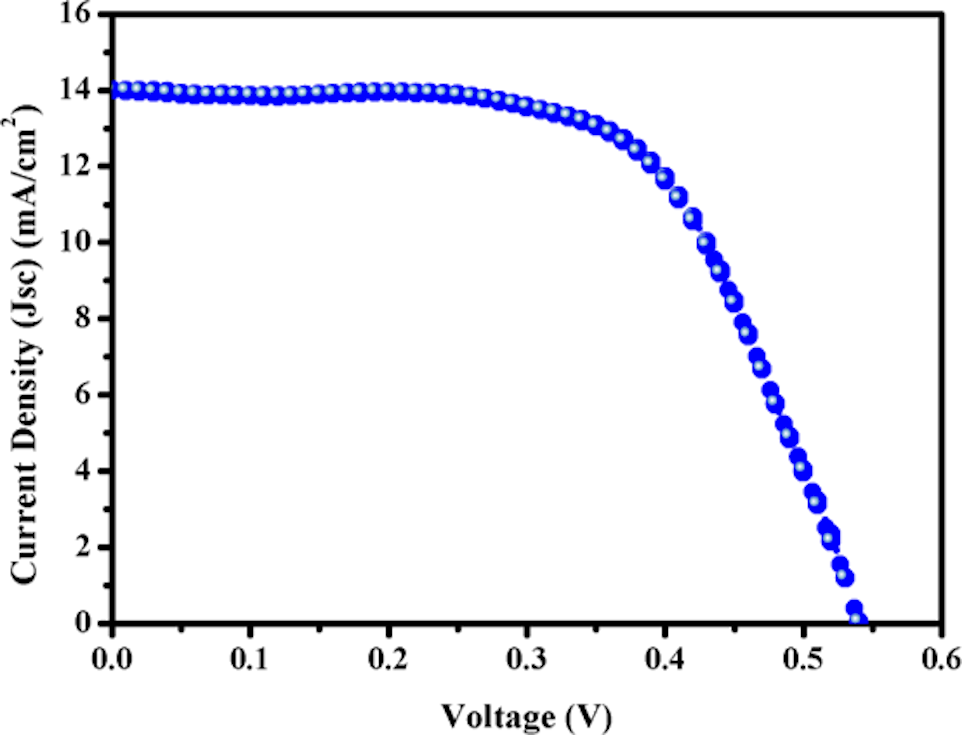
*J*–*V* curve of the fabricated QDSCs.

The *J*–*V* curves of the AZGSSe/TiO_2_ photoanode-based QDSCs are displayed in Figure 8 and the corresponding photovoltaic parameters *J*_sc_, *V*_oc_, and FF are 14.20 mA/cm^2^, 0.54 V, and 0.64, respectively. Its PCE was calculated to be 4.91%. This PCE is greater than that of the Ag-In-S QD-sensitized QDSCs (2.39%) [15], Zn-Ag-In-S QD-sensitized QDSCs (4.45%) [15], and Zn-Ag-In-Se QD-sensitized QDSCs (3.57%) [16]. This is due to the wide-range light-harvesting capacity of AZGSSe QDs and the enhanced electron transfer from the conduction band of AZGSSe QDs to TiO_2_ NFs. The values of *R*_s_ and τ_s_ and the photovoltaic parameters of the fabricated QDSC in comparison with those of a Zn-Ag-In-S QD-sensitized QDSC and a Zn-Ag-In-Se QD-sensitized QDSC are given in Table 2.

**Table 2:** The values of *R*_s_ and τ_s_ and photovoltaic parameters of the synthesized QDSC in comparison with those of a Zn-Ag-In-S QD-sensitized QDSC and a Zn-Ag-In-Se QD-sensitized QDSC.

QDs	*R*_2_ (Ω)	τ_s_ (ns)	*J*_sc_ (mA/cm^2^)	*V*_oc_ (V)	FF	PCE %	Ref

Zn-Ag-In-S	164.6	284.76	13.96	0.54	0.59	4.50	[15]
Zn-Ag-In-Se	46.87	41.40	16.03	0.38	0.58	3.57	[16]
Ag-Zn-Ga-S-Se	26.78	18.92	14.20	0.54	0.64	4.91	this work

## Conclusion

Ag-Zn-Ga-S-Se alloyed QDs with a diameter of 5.11 nm were synthesized by a hot injection method. From the EDX analysis, its stoichiometric ratio was found to be 1:1:1:1.5:1.5. The oxidation sate of the elements in the synthesized QDs are examined to be +1, +2, +3, −2 and −2 for, respectively, Ag, Zn, Ga, S, and Se. Optical analysis revealed the excellent optical properties of the synthesized QDs. Because of their good NIR light absorption, the synthesized QDs were loaded onto TiO_2_ NFs, which then formed the photoanode of a QDSC. The fabricated QDSC shows an improved PCE of 4.91%. This is due to the enormous light-harvesting capacity of AZGSSe QDs and the enhanced electron transfer from AZGSSe QDs to TiO_2_ NFs.
